# The Effect of Preceding Self-Control on Prosocial Behaviors: The Moderating Role of Awe

**DOI:** 10.3389/fpsyg.2019.00682

**Published:** 2019-03-26

**Authors:** Jin Li, Anke Li, Yu Sun, Hui’ e Li, Lei Liu, Youlong Zhan, Wei Fan, Yiping Zhong

**Affiliations:** ^1^Department of Psychology, Hunan Normal University, Changsha, China; ^2^Cognition and Human Behavior Key Laboratory of Hunan Province, Changsha, China; ^3^School of Psychological and Cognitive Sciences, Peking University, Beijing, China

**Keywords:** self-control, prosocial behavior, awe, protective inhibition of self-regulation and motivation model, moderating role

## Abstract

The exertion of self-control is known to result in subsequent detrimental effects on prosocial behaviors. Moreover, certain studies have demonstrated that positive emotions could drive people to allocate more attentional resources for conducting prosocial behaviors. However, whether and how awe – one important type of positive incidental emotion – moderates the effect of exerting self-control on subsequent prosocial behaviors remains unclear yet. The anonymous economic dictator game is an effective index of prosocial behaviors. We examined the influence of exerting self-control on prosocial behavior and the moderating role of awe on the effect of exerting self-control on prosocial behaviors in two experiments (*N* = 280). We adopted the incongruent Stroop task to induce the exertion of self-control and participants were required to allocate money to others in the anonymous dictator game (*Experiment 1*). We used the narrative recall task paradigm to elicit the emotion of awe during the interval between Stroop tasks and the dictator game (*Experiment 2*). Results indicated that the exertion of self-control was detrimental to prosocial behaviors and awe weakened the detrimental effects of exerting self-control on prosocial behavior. We interpreted these results in terms of the protective inhibition of self-regulation and motivation (PRISM) model.

## Introduction

Self-control is an important ability that allows people to manage their thoughts, feelings, and actions, ranging from executive cognitive functions like attention control to higher-order processes such as affect-regulation (Gendolla et al., 2015). Experimental evidence has demonstrated that people are less likely to behave prosocially after an initial act requiring self-control (Dewall et al., 2007; Mead et al., 2009; Gino et al., 2011), presumably because they needed to override their automatic selfish impulses by exerting self-control (Dewall et al., 2008; Joosten et al., 2015). For example, Dewall et al. (2008) showed that people reported being less likely to help other people even in imagined scenarios after they completed the incongruent Stroop task. In contrast, people who did not conduct an initial act of self-control demonstrated the highest executive capacity to control selfish impulses and behave prosocially to others (Gino et al., 2011; Kai et al., 2014; Kai, 2018). Therefore, it is possible that performing a task involving self-control would subsequently decrease prosocial behaviors.

A body of literature indicates that positive emotions may moderate the negative relationship between exerting self-control and prosocial behaviors (Tice et al., 2007; Ren et al., 2010). In particular, after performing an initial self-control task, positive emotions such as happiness restore the subsequent performance of self-control. Previous studies have indicated that awe is an important and special incidental positive emotion (Vaillant, 2008; Dong et al., 2013), which is defined as an emotional response to perceptually vast stimuli that overwhelm an individual’s current mental structures (Mossman, 2007). Thus, awe might be distinct from other positive emotions. However, whether awe exerts a special moderating effect on the negative relationship between exerting self-control and prosocial behaviors remains unknown.

We found that the protective inhibition of self-regulation and motivation (PRISM) model might be a reasonable and appropriate model for predicting the moderating effects of the awe on the relationship between exerting self-control and prosocial behaviors. Tops et al. (2015) developed the PRISM, which postulates that a continuous cost-benefit analysis, integrating the history of preceding performance and its efficiency, inhibits task engagement to prospectively prevent adverse health consequences from inefficient or excessive performance of tasks that require self-control. Unlike the previous studies suggesting that it is implausible that self-control tasks of modest duration deplete (e.g., physiological) resources, as suggested by the strength model (Kurzban, 2010; Beedie and Lane, 2012; Inzlicht et al., 2014). Here, self-control is defined by sustained vigilance to prevent attentional lapses and undesired habitual or hedonically driven responses. The exertion of self-control entails a challenge to the homeostasis, potential physiological costs, and eventually health consequences because of concurrent sympathetic activation (Sterzer and Kleinschmidt, 2010). The PRISM model holds that the accumulation of potentially costly physiological activation upregulates a protective mechanism that increases resistance against self-control by increasing subjective effortfulness (Tops et al., 2016). In other words, the process of PRISM increases resistance to focusing attention and self-control resources for taking on subsequent self-control tasks. The PRISM explains the results of recent re-analyses of the multi-lab study by Hagger and Chatzisarantis (2016), which showed that the effect of an initial self-control task on the performance in a subsequent self-control task operates through the mediation of self-rated effort, difficulty, frustration, and fatigue (Drummond and Philipp, 2017), and that self-rated effortfulness of the initial task predicts subsequent performance impairments (Dang et al., 2017). Mediation through subjective effortfulness in the context of a task requiring an overriding dominant response indicates that the need to exert high levels of attentional effort in self-control leads to increased PRISM. The PRISM is a sub-theory of the broader predictive and reactive control systems (PARCS) theory, which integrates evidence that the reactive (vigilance) system takes control in unpredictable situations when internal models established by previous learning cannot predict the amount of resources needed to successfully cope with a performance challenge (Tops, 2017). Therefore, this system will over-mobilize resources (“just in case”) through the activation of the sympathetic nervous system and hypothalamic-pituitary-adrenal axis (e.g., cortisol). The system is activated when feedback-guided, momentary control of behavior is needed, which is often the case in novel situations or during non-adapted task performance (Nicola, 2010), and in the case of non-habituated, urgent and emergency situations that override dominant responses, as well as when vigilance is required. PRISM, which is indexed by the length (Hagger et al., 2010) and intensity of activation in this system, temporarily increases resistance against performing tasks that activate the system. By contrast, predictive control guided by internal models established by previous learning takes over in less urgent situations and after adaptation to tasks. Predictive control produces access to long-term goals and efficient regulation of physiology and homeostasis including recuperation. In other words, predictive control decreases PRISM. Consistent with this notion, researches have shown that adapting to either the first (Dang et al., 2013) or the second task (Barutchu et al., 2013) removes the performance deterioration effect without rest or additional motivation.

Certain positive emotions and practices might also be effective in reducing PRISM because they increase or reflect predictive control (Tops et al., 2013). For instance, meditative practices such as mindfulness and praying, which are associated with a sense of purpose and decreased urgency (MacLeod, 2017), restore the performance of self-control after preceding use of self-control (Friese et al., 2012, 2014). As awe is associated with a sense of purpose and decreased urgency (see below), we propose that awe might reduce PRISM because of an association of awe with predictive control, although this possibility has not yet been investigated in this context. Rudd et al. (2012) reported that awe might increase the perception that time is plentiful and therefore reduce impatience or the sense of urgency. This is particularly relevant because perceived time availability appears to be associated with predictive control and long-term goals. If we perceive time to be abundant, we are more likely to eat healthily, help other people in distress, and engage in leisure activities (Rudd et al., 2012). However, there is no direct and explicit experimental evidence based on the PRISM model regarding the moderating role of awe in the relationship between the exertion of self-control and prosocial behaviors. Consequently, it is the goal of this study to examine whether the emotion of awe can moderate the relationship between preceding exertion of self-control and prosocial behaviors.

## Overview of the Current Study

Past studies have examined prosocial behaviors by adopting different paradigms. Among these, the anonymous economic dictator game has been widely used in many studies (Bolton et al., 1998; Shariff and Norenzayan, 2007; Xu et al., 2012; Zhao et al., 2017). The results of these studies have suggested that the anonymous economic dictator game is suitable for investigating prosocial behaviors because this task provides an honest indication of prosocial behaviors without the artifacts caused by participants’ need to manage impressions (Shariff and Norenzayan, 2007). In the economic dictator game, participants have the opportunity to allocate a certain amount of money to another participant (described as a stranger to him/her) according to their wishes. Therefore, we adopted the economic dictator game paradigm in the present study to investigate the relationship between the exertion of self-control and subsequent prosocial behaviors.

We conducted two experiments to investigate two hypotheses: In Experiment 1, we adopted two types of Stroop tasks: The incongruent Stroop task condition manipulated the exertion of self-control, whereas the congruent Stroop task condition was the control condition. The anonymous economic dictator game was used to investigate the hypothesis that preceding self-control subsequently hinders prosocial behavior. Subsequently, in Experiment 2, we investigated the hypothesis that awe counteracts the adverse effect of preceding self-control on prosocial behavior.

Prior to each experiment of this study, all the participants provided written informed consent according to the Declaration of Helsinki, after they fully understood the content of the experiments. Participants completed the task and received a monetary payment according to the consequences of the dictator game. The experiments were approved by the Institutional Review Board of Hunan Normal University, Department of Psychology.

## Experiment 1

In Experiment 1, we asked participants to complete the incongruent Stroop task to induce the exertion of self-control, and then we adopted the anonymous economic dictator game to examine the participants’ prosocial behavior. Based on previous literature described above, we hypothesized that the exertion of self-control would decrease prosocial behavior in the incongruent Stroop task condition, which would be demonstrated by participants allocating less money to the other person in the incongruent Stroop task condition compared to the congruent Stroop task condition.

## Materials and Methods

### Participants and Design

Eighty healthy undergraduates (37 men; *M*_age_ = 21.70 years, *SD* = 1.73) from Hunan Normal University participated in the experiment. All the participants were randomly assigned to either the incongruent Stroop task (40 participants, 19 men) or the congruent Stroop task conditions (40 participants, 18 men). They completed the experimental tasks and received a monetary reward according to the consequences of the dictator game.

### Procedure

#### Manipulating the Exertion of Self-Control

**FIGURE 1:**
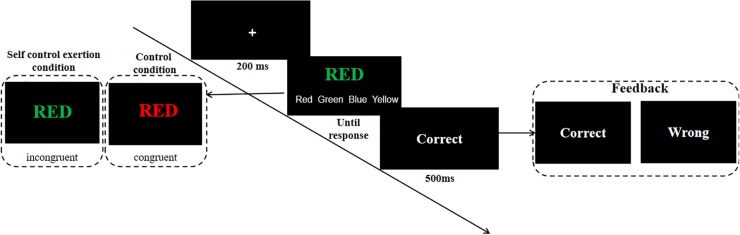
An illustration of a single trial in the Stroop task in Experiment 1 and 2. Each trial began with a fixation cross. Then participants were asked to indicate the displayed color of words, while ignoring the meaning of the words (i.e., RED, GREEN, BLUE, YELLOW). All the stimuli in the congruent Stroop task (control condition) were congruent, such that the meaning of words and the displayed colors were matching (e.g., “GREEN” was displayed in green). In contrast, in the incongruent Stroop task condition, all stimuli used in the Stroop task were incongruent, such that words meanings and displayed colors did not match (e.g., “BLUE” was displayed in yellow). Participants were required to recognize the color of the words without regard to the meaning of the words and clicked a mouse on one of four buttons that were displayed below the stimulus area of on the screen, which were labeled “Red,” “Green,” “Blue,” or “Yellow” written in white (randomized for each participant).

We adopted the modified color Stroop task to manipulate the exertion of self-control based on the study by Singh and Göritz (2018). A recent meta-analysis has suggested that performing the Stroop task is effective for reducing subsequent performance on another self-control task (Dang, 2018). In the Stroop task, participants were asked to indicate the displayed color of words, while ignoring the meaning of the words (i.e., RED, GREEN, BLUE, YELLOW). All the stimuli in the congruent Stroop task (control condition) were congruent, such that the meaning of words and the displayed colors were matching (e.g., “GREEN” was displayed in green). In contrast, in the incongruent Stroop task condition, all stimuli used in the Stroop task were incongruent, such that words meanings and displayed colors did not match (e.g., “BLUE” was displayed in yellow). The congruent and incongruent Stroop task conditions consisted of 100 trials with an equal distribution of colors. The word in each trial was displayed until the participant responded. Participants were required to recognize the color of the words without regard to the meaning of the words and clicked a mouse on one of four buttons that were displayed below the stimulus area of on the screen, which were labeled “Red,” “Green,” “Blue,” or “Yellow” written in white (randomized for each participant). Then, feedback on whether the response was correct or not was displayed for 500 ms (see Figure 1). After completing the task, the participants responded to the manipulation check that assessed the task difficulty. Participants also indicated the extent to which they fought against the urge to select the dominant responses while conducting the task by responding to a 7-point scale (Boucher and Kofos, 2012; Tops, 2017) ranging from 1 (*disagree strongly*) to 7 (*agree strongly*). Then, the participants responded to an emotional assessment in which they responded the extent to which they felt happy/proud/sad/angry/disgusted/or awe, after completing the Stroop task. Responses were made using a 7-point scale (Piff et al., 2015) ranging from 1 (do not feel at all) to 7 (feel strongly).

#### Assessment of Prosocial Behavior

Following the above task, each participant in both conditions performed an anonymous version of the economic dictator game (Elizabeth et al., 1994; Shariff and Norenzayan, 2007), with a confederate who was a virtual stranger. The game consisted of eight rounds in which the participants had eight opportunities to allocate as much money as they wanted. In each round, they were given the following instructions: “*You have been chosen as the benefactor in an economic decision-making task. You will find 10 Yuan (a unit in renminbi, RMB). Your role is to take and keep as much of this money as you would like, knowing that, however, much you leave, if any, it would be given to the next participant whom you will never know, to keep as his or her own.*”

## Results and Discussion

### Self-Control Exertion Manipulation Checks

Participants in the incongruent Stroop task condition indicated that the task was more difficult (*M* = 2.65, *SD* = 0.58) than in the congruent Stroop task condition (i.e., control condition) (*M* = 1.70, *SD* = 0.65), *t*(78) = 6.91, *p* < 0.001, *d* = 0.74. Moreover, participants in the incongruent Stroop task condition reported that they were fighting against an urge to select dominant responses (*M* = 3.30, *SD* = 0.88) more than in the control condition (*M* = 1.80, *SD* = 0.65), *t*(78) = 8.66, *p* < 0.001, *d* = 1.92. Tops (2017) suggested that the effort and the difficulty of tasks required to override a dominant response indicate the need to exert a high level of effort for attentional self-control, which is associated with increased PRISM.

### Emotions Checks

There were no significant differences in the seven emotions between the two Stroop task conditions. Table 1 shows the *t*-values between the two experimental Stroop task conditions.

**Table 1:** The *t*-values for emotions between two experimental Stroop task conditions.

	Awe	Fear	Pride	Anger	Sad	Disgust	Happy
*t*	0.25	0.97	0.39	0.52	0.66	0.17	1.21
*p*	0.87	0.64	0.82	0.75	0.70	0.91	0.53


### The Results in the Anonymous Economic Dictator Game

**FIGURE 2:**
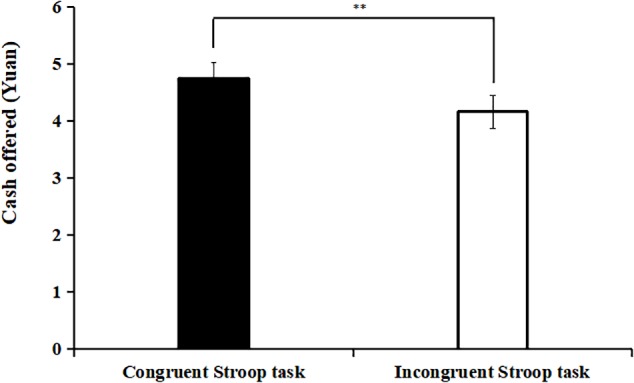
The mean money allocated to the other person in the incongruent Stroop task condition or congruent Stroop task condition in Experiment 1. ^∗∗^*p* < 0.001.

We analyzed the mean amount of money allocated to the other person across the eight rounds by each participant, by conducting an Independent sample *t*-test to examine the effect of the preceding exertion of self-control on the money that was allocated to the other person between two conditions. Results indicated that participants in the incongruent Stroop task condition (*M* = 4.16, *SD* = 0.73) allocated less money to the other person than in the congruent Stroop task condition (*M* = 4.74, *SD* = 0.77), *t*(78) = -3.41, *p* < 0.001, *d* = 0.33, suggesting that exerting self-control subsequently decreased prosocial behavior (see Figure 2).

Our hypothesis was that the exertion of self-control would decrease an individual’s prosocial behavior. The results of Experiment 1 indicated that participants allocated less money to the other person after they completed the incongruent Stroop task compared to the control condition (the congruent Stroop task). This finding is consistent with previous studies (Crockett et al., 2010; Piff et al., 2015; Fei et al., 2016; Nolet et al., 2018). According to the PRISM model, performing a self-control task temporarily increases effortfulness and resistance against a subsequent exertion of self-control (Tops et al., 2015). The increased resistance against exerting self-control might have decreased prosocial behaviors (Dewall et al., 2008; Joosten et al., 2015).

Past studies have demonstrated that evoking positive emotions such as happiness could restore an individuals’ performance of self-control (Ren et al., 2010). Awe is known to be a positive and spiritual emotion that shifts attention away from immediate concerns and urgencies caused by self-interest (Prade and Saroglou, 2016; Jiang et al., 2018), perhaps by shifting attention toward long-term goals and purposes based on the PRISM model. Although studies have shown a relationship between awe and prosocial behaviors, little is known about whether and how awe directly moderates the relationship between the exertion of self-control and prosocial behaviors.

## Experiment 2

The purpose of Experiment 2 was twofold, firstly, we aimed to replicate the effect of exerting self-control on prosocial behavior, and secondly and more importantly, we wanted to examine whether awe could moderate the effects of exerting self-control on prosocial behavior. We adopted the same paradigms in Experiment 2 as we did in Experiment 1 but added a new condition of awe vs. no-awe.

## Materials and Methods

### Participants and Design

We recruited two hundreds healthy undergraduates (107 men; *M*_age_ = 20.42 years, *SD* = 3.17) from Hunan Normal University to participate in the experiment. They were randomly assigned to four experimental conditions (i.e., 50 participants in the incongruent Stroop task condition-awe prime; 50 participants in the incongruent Stroop task condition – no-awe prime; 50 participants in the congruent Stroop task condition- non-awe prime; 50 participants in the congruent Stroop task condition-awe prime). Thus, the experiment consisted of a 2 (Self-control exertion manipulation: taking the congruent Stroop task vs. taking the incongruent Stroop task) × 2 (Emotion prime types: awe prime vs. no-awe prime) between-subject design.

### Materials

We used a narrative recall task paradigm as an awe prime or a no-awe prime, by adopting the materials used by Piff et al. (2015). The participants of this study were recruited in China and were Chinese language speakers. Therefore, prior to the main experiment, we translated the materials into Chinese. Then, we required 120 undergraduates that did not participate in the Experiment to rate the effectiveness of the material. For the materials in the awe prime condition, 60 undergraduates read the following instructions: “*Please take a few minutes to think about a particular time, fairly recently, when you encountered a natural scene that caused you to feel awe. This might have been a sunset, a view from a high place, or any other time you were in a natural setting that you felt was beautiful.*” Half of the 60 undergraduates read the no-awe prime materials: “*Please take a few minutes to think about something you did fairly recently. This might have been riding a bike, studying for a test, or any other thing that happened during your day.*” After reading the instructions, all the participants wrote down sentences describing the experience in as much detail as possible (Jiang et al., 2018) and rated how they experienced the emotions (including Happiness/Pride/Sadness/Anger/Disgust/Fear/Awe) on a 7-point Likert Scale (Piff et al., 2015).

We conducted an Independent sample-*t*-test to examine the effectiveness of the materials. The rating results indicated that except for awe [*t*(118) = 2.45, *p* < 0.001, *d* = 0.62], there were no differences between the awe prime and non-awe prime conditions {Fear [*t*(118) = 0.61], Disgust [*t*(198) = 0.06], Anger [*t*(198) = 0.18], Sadness [*t*(198) = 0.52], Pride [*t*(198) = 0.12], and Happiness [*t*(198) = 0.14] (*p*s > 0.05)}. Therefore, we concluded that the material that we developed in the Chinese language could effectively evoke awe.

### Procedure

The main section of Experiment 2 consisted of three parts. *In the first part of* Experiment 2, participants were provided with different manipulations for exerting self-control. We adopted the same paradigms in Experiment 2 as we did in Experiment 1. *In the second part of* Experiment 2, participants in awe prime, incongruent and congruent Stroop task conditions completed the awe-evoking task by reading the awe priming material and writing sentences describing their awe experience in as much detail as possible. Participants in no-awe prime, congruent and incongruent Stroop task conditions completed the no-awe priming task. Following this, each participant rated his or her emotional experiences including Happiness, Pride, Sadness, Anger, Disgust, Fear, and Awe on the 7-point Likert scale. Finally, *in the third part of* Experiment 2, each participant conducted the economic dictator game that we used in Experiment 1.

## Results and Discussion

### Self-Control Exertion Manipulation Checks

We conducted a 2 (Self-control exertion manipulation: the congruent Stroop task vs. the incongruent Stroop task) × 2 (Emotion prime types: awe prime vs. no-awe prime) ANOVA on exertion of self-control manipulation measurement. We observed that there was a significant main effect in the task difficulty rating, *F*(1,196) = 7.44, *p* < 0.001, $\eta_{p}^{2}$ = 0.37, suggesting that participants in the incongruent Stroop task condition thought the task was more difficult (*M* = 3.05, *SD* = 0.69) than in the congruent Stroop task condition (*M* = 2.10, *SD* = 0.47). There was a significant main effect in fighting against the urge to select dominant responses, *F*(1,196) = 9.14, *p* < 0.001, $\eta_{p}^{2}$ = 0.51, demonstrating that participants in the incongruent Stroop task condition reported that they were fighting the urge (*M* = 4.12, *SD* = 0.65) more than in the congruent Stroop task condition (*M* = 2.03, *SD* = 0.70). There were no other effects of the self-control exertion manipulation checks (*F*s < 1, *p*s > 0.05). These rating results indicated that the self-control manipulation was successful.

### Awe Manipulation Checks

We observed a main effect of Emotion prime types on Awe rating, *F*(1,196) = 11.62, *p* < 0.001, $\eta_{p}^{2}$ = 0.59, suggesting that participants in the awe prime condition indeed felt more awe (*M* = 4.97, *SD* = 0.41) than in the non-awe prime condition (*M* = 0.82, *SD* = 0.15).

### The Results in the Anonymous Dictator Game

**FIGURE 3:**
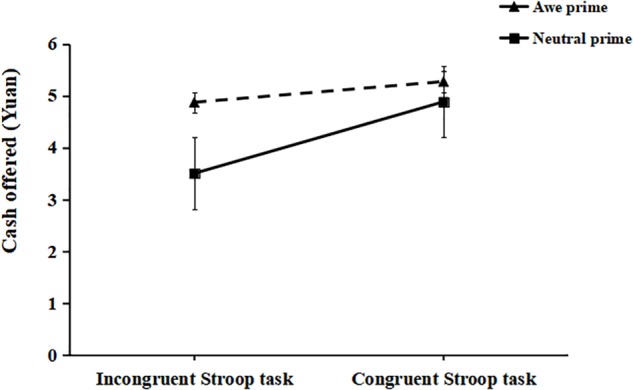
The moderating effect of awe on the relationship between the exertion of self-control and the mean money allocated to the other in the dictator game of Experiment 2.

A 2 (Self-control exertion manipulation: the congruent Stroop task vs. the incongruent Stroop task) × 2 (Emotion prime types: awe prime vs. no-awe prime) ANOVA was conducted on the money amounts that the participants allocated to the stranger. The ANOVA indicated a main effect of manipulating the exertion of Self-control *F*(1,196) = 46.65, *p* < 0.001, $\eta_{p}^{2}$ = 0.59, which demonstrated that the participants in the incongruent Stroop task condition allocated less money to the other person (*M* = 4.19, *SD* = 0.96) than in the congruent Stroop task condition (*M* = 5.08, *SD* = 0.86). There was also a main effect of Emotion Prime types, *F*(1,196) = 47.18, *p* < 0.001, $\eta_{p}^{2}$ = 0.61, indicating that participants in the awe prime condition (*M* = 5.07, *SD* = 0.91) allocated more money to the other person than in the no-awe prime condition (*M* = 4.20, *SD* = 0.92). Importantly, there was a significant interaction effect of the manipulation of exerting self-control × Emotion prime types, *F*(1,196) = 14.20, *p* < 0.001, $\eta_{p}^{2}$ = 0.32. Simple effect analysis showed that there was a significant difference between the awe prime (*M* = 4.87, *SD* = 0.99) and no-awe prime conditions (*M* = 3.50, *SD* = 0.93) in the incongruent Stroop task condition, *F*(1,196) = 56.58, *p* < 0.001, $\eta_{p}^{2}$ = 0.69. By contrast, the difference between the awe prime condition and non-awe prime condition was not significant in the congruent Stroop task condition, *F*(1,196) = 4.81, *p* = 0.059, $\eta_{p}^{2}$ = 0.16 (see Figure 3).

### The Moderating Effect of Self-Reported Awe

**FIGURE 4:**
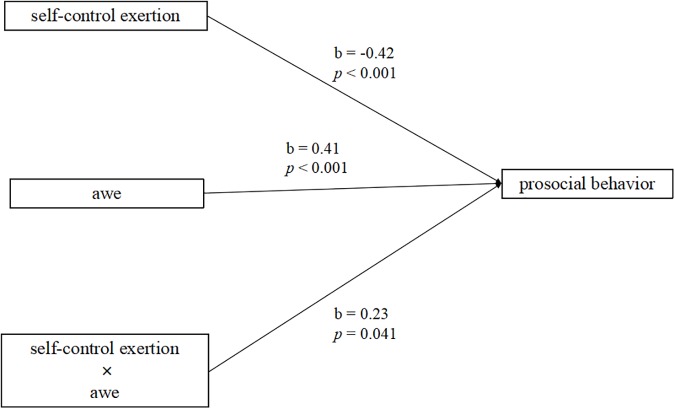
Moderated model: The awe moderates the effect of the exertion of self-control on the prosocial behavior.

A moderation analysis was conducted to examine whether the self-reported awe moderated the negative relationship between the exertion of self-control and prosociality. Based on the study by Boucher and Kofos (2012), we selected *the incongruent Stroop task-awe prime* group and *the congruent Stroop task-awe prime* group (one hundred participants in total) to examine whether the emotion of awe moderated the effect of the exertion of self-control on the performance in the anonymous dictator game. The self-control manipulation conditions (0 = *the incongruent Stroop task*, 1 = *the congruent Stroop task*) were dummy-coded in this analysis. The scores of the emotion of awe were also processed by mean-centering based on a previous study (Jie, 2015). Hierarchical multiple regression analysis revealed that the exertion of self-control was significantly and negatively related to the performance in the dictator game, *b* = -0.42, *t* = 3.12, *p* < 0.001, and the score for the emotion of awe was also significantly and positively related to the performance in the economic dictator game, *b* = 0.41, *t* = 3.10, *p* < 0.001. The interaction between the exertion of self-control and awe was significantly related to the performance in the dictator game, *b* = 0.23, *t* = 2.09, *p* = 0.041, the adjusted R^2^ was 0.03. This suggested that experiencing awe enhanced prosocial behavior, and it moderated the relationship between the exertion of self-control and the performance of prosocial behavior in the economic dictator game (see Figure 4).

Experiment 2 provided evidence supporting the hypothesis that awe moderates the negative relationship between the exertion of self-control and prosocial behavior. The participants in the incongruent Stroop task condition that were primed with awe allocated more money to another person compared to participants in the no-awe prime condition. Furthermore, the analyses showed that self-reported awe partly moderated the effect of the exertion of self-control on prosocial behavior.

## General Discussion

Our findings provide experimental evidence that awe moderates the negative effect of the exertion of self-control on prosocial behaviors. Specifically, the results of Experiment 1 supported the notion that the exertion of self-control decreases prosocial behavior, and the results of Experiment 2 showed that awe moderated the negative relationship between the exertion of self-control and prosocial behavior, such that the detrimental effect of the exertion of self-control was buffered by recalling an experience of awe.

### Exertion of Self-Control Decreases Prosocial Behavior

Both Experiments 1 and 2 supported the hypothesis that the prosocial behavior of participants would diminish after completing the incongruent Stroop task. In terms of PRISM, exerting self-control might increase resistance against taking on further self-control tasks. The anonymous dictator game, which measured the prosocial behavior, was regarded in this study as a task that requires the exertion of self-control to suppress selfish impulses (Shariff and Norenzayan, 2007; Xu et al., 2012; Ainsworth et al., 2014). This type of task was expected to be negatively affected by PRISM. Many studies have suggested that being prosocial to others is consistent with social norms and standards (Schultz et al., 2007; House, 2017; Paulus, 2017). Suppressing selfish impulses, such as seeking immediate gratification, is known to require the exertion of self-control because self-control is needed to bring behavior into line with social norms (Gailliot et al., 2012). Conversely, if PRISM increased resistance against exerting self-control, individuals would be less likely to comply with social norms, and they would seek immediate gratification that satisfies their selfish impulses (Baumeister and Vohs, 2016).

### The Moderating Effect of Awe

In Experiment 2, we observed that participants in the awe prime condition allocated more money to the other person than in the no-awe prime condition, suggesting that awe indeed promoted prosocial behavior. This finding is consistent with previous literature (Piff et al., 2015; Ying et al., 2016; Bai et al., 2017). Awe-triggering stimuli such as viewing the ocean, an enormous forest, high buildings or mountains, diminish the sense of self (Lorkowski and Kreinovich, 2015; Piff et al., 2015; Prade and Saroglou, 2016; Stellar et al., 2017). Awe orients individuals to lower their self-interests and immediate concerns and highlight the interest in others by diminishing the attentional importance of the self (Keltner et al., 2014; Bai et al., 2017). Therefore, in this study, the experience of awe might have promoted prosocial behavior in the economic dictator game.

Crucially, awe plays a moderating role in the negative relationship between the exertion of self-control and prosocial behavior. The emotion of awe might reduce PRISM because it shifts people from reactive (vigilant) control, which is regulated and limited by PRISM because of its physiological consequences, toward predictive control. In turn, predictive control is associated with access to long-term goals and internal models, and with effective and recuperative regulation of physiology and homeostasis (Tops, 2017). Consistent with the assumption that awe facilitates predictive control, Rudd et al. (2012) for example, demonstrated that awe might increase the perception that time is plentiful and therefore reduces impatience and the sense of urgency. Awe also triggers a desire in people to expand their internal models (Keltner and Haidt, 2003) and people are motivated to acquire new knowledge when the time feels expansive (Carstensen et al., 1999). If we perceive time to be abundant, we are more likely to eat healthily, help others in distress and engage in leisure activities (Rudd et al., 2012). Awe shifts attention away from immediate concerns about, and urgency of, self-interests, perhaps by shifting toward long-term goals and purposes. Hence, awe may reduce PRISM similar to meditative practices that seem to facilitate predictive control as suggested by their association with a sense of purpose and decreased urgency (Tops et al., 2014; MacLeod, 2017). Specifically, mindfulness and praying restored self-control performance that was reduced following the exertion of preceding self-control (Friese et al., 2012, 2014). The effect of awe to shift away from reactive control and allow for recuperation and resource building (e.g., by extending internal models) reduces PRISM by impacting on the underlying cost-benefit analysis for subsequent self-control. In addition, we observed that awe only has a partially moderating effect on the relationship between the exertion of self-control and prosocial behavior. This could be because not all participants were equally successful in inducing awe by recall. In the incongruent Stroop condition, participants that were less successful in experiencing awe reported less awe and behaved less prosocially.

In terms of the motivation theory of Inzlicht et al. (2014), It could be argued that the Stroop task and the dictator game constitute “have-to” tasks whereas the awe task constitutes a “want-to” task. Hence, it is possible that the induction of awe after the Stroop task restored the balance between “have-to” and “want-to” tasks, facilitating the recovery of performance in the subsequent “have-to” task. Future research should replicate the present findings and discriminate between these theories by analyzing the precise factors that make a task potentially effective for decreasing the performance on a subsequent task, factors that make a task sensitive to this effect, and factors that make a task an effective moderator (e.g., restorer of performance in the subsequent task). Furthermore, it would be informative to assess subjective effortfulness and resistance at the initiation of the Stroop tasks to test predictions of the PRISM model, as well as to measure the activation of the autonomic nervous system.

## Conclusion

Our findings in the present study indicated that the exertion of self-control hindered prosocial behavior in the anonymous economic dictator game. Moreover, the emotion of awe could counteract the adverse effect of the exertion of self-control on prosocial behavior.

## Ethics Statement

Each participant signed an informed consent form. The Ethics Committee of Hunan Normal University approved this study.

## Author Contributions

JL, AL, and YpZ designed the experiments. JL, WF, YS, and HL recruited participants and collected the data. JL, LL, and YlZ performed the data analyses. JL and YpZ wrote the manuscript.

## Conflict of Interest Statement

The authors declare that the research was conducted in the absence of any commercial or financial relationships that could be construed as a potential conflict of interest.
